# Non-operative management for osteochondral lesions of the talus: a systematic review of treatment modalities, clinical- and radiological outcomes

**DOI:** 10.1007/s00167-023-07408-w

**Published:** 2023-04-16

**Authors:** Tristan M. F. Buck, Kenny Lauf, Jari Dahmen, J. Nienke Altink, Sjoerd A. S. Stufkens, Gino M. M. J. Kerkhoffs

**Affiliations:** 1 Amsterdam UMC location University of Amsterdam, Department of Orthopedic Surgery and Sports Medicine, Amsterdam, The Netherlands; 2 Amsterdam Movement Sciences, Musculoskeletal Health, Amsterdam, The Netherlands; 3 Academic Center for Evidence-Based Sports Medicine (ACES), Amsterdam, The Netherlands; 4 Amsterdam Collaboration on Health & Safety in Sports (ACHSS), IOC Research Center, Amsterdam, The Netherlands

**Keywords:** Osteochondral lesions of the talus, OLT, Conservative, Non-operative, Ankle, Cartilage

## Abstract

**Purpose:**

The purpose of the present study was to assess the overall clinical success rate of non-operative management for osteochondral lesions of the talus (OLT).

**Methods:**

A literature search was conducted in the PubMed (MEDLINE), COCHRANE and EMBASE (Ovid) databases. Clinical success rates per separate study were calculated at the latest moment of follow-up and were defined as successful when a good or excellent clinical result at follow-up was reported in a qualitative manner or when a post-operative American Orthopaedic Foot and Ankle Society (AOFAS) score at or above 80 was reached. When clinical outcomes were based on other clinical scoring systems, outcomes reported as good or excellent were considered as clinical success. Studies methodologically eligible for a simplified pooling method were combined to calculate an overall pooled clinical success rate. Radiological changes over the course of conservative treatment were assessed either considering local OLT changes and/or overall ankle joint changes.

**Results:**

Thirty articles were included, including an overall of 868 patients. The median follow-up of the included studies was 37 months (range: 3–288 months). A simplified pooling method was possible among 16 studies and yielded an overall pooled clinical success rate of 45% (95% CI 40–50%). As assessed with plain radiographs, progression of ankle joint osteoarthritis was observed in of 9% (95% CI 6–14%) of the patients. As assessed through a Computed Tomography (CT) scan, focal OLT deterioration was observed in 11% (95% CI 7–18%) of the patients. As assessed with a Magnetic Resonance Imaging (MRI) scan, focal OLT deterioration was observed in 12% (95% CI 6–24%) of the patients. An unchanged lesion was detected on plain radiographs in 53% (48/91; CI 43–63%), 76% (99/131; 95% CI 68–82%) on a CT scan and on MRI in 84% (42/50; 95% CI 71–92%) of the patients.

**Conclusion:**

The current literature on non-operative management of OLTs is scarce and heterogeneous on indication and type of treatment. Promising clinical results are presented but need to interpreted with caution due to the heterogeneity in indication, duration and type of treatment. Further studies need to focus on specific types on conservative management, indications and its results.

**Level of evidence:**

Systematic review, Level IV.

**Supplementary Information:**

The online version contains supplementary material available at 10.1007/s00167-023-07408-w.

## Introduction

Osteochondral lesions of the talus (OLTs) are focal problems of the cartilage and its subchondral bone [10, 25]. The first treatment in line for the specific injury is a non-operative protocol potentially consisting of different types of subtypes of non-operative treatment options [12]. A surgical intervention can be needed in case of persistent and restrictive complaints after at least 6 months of non-operative treatment protocol.

To date, the most comprehensive study concerning non-operative treatment available is a systematic review from Zengerink et al. [44]. Despite the clear oversight of outcomes, this study included articles published up to the year of 2006 and an update of outcomes will add value to daily practice. Furthermore, radiological outcomes were not assessed and further sub-specify the clinical efficacy of the different subtypes of non-operative treatment protocols beyond rest and casting.

Consequently, it is currently unclear how effective non-operative therapy is for osteochondral lesions of the talus (OLTs) and which sub-type of non-operative therapy is most effective. It is therefore the purpose of the this study to assess the overall clinical success rate and associated radiological progress of focal or generalized joint degenerative changes after non-operative management for OLTs and to analyze different subtypes of non-operative management if possible. Our secondary hypothesis is that non-operative management yields an overall successful clinical result in approximately half of the patients.

## Materials and methods

This review was performed according to the PRISMA guidelines and was prospectively registered in the Prospero database with registration number CRD42018114667 [9].

### Search strategy

An electronic literature search was performed—the specific search strategy is presented in Appendix 1. After the first search, title and abstracts were screened for eligibility based on the inclusion- and exclusion criteria (Table 1).

**Table 1:** Inclusion- and exclusion criteria

Inclusion criteria	Exclusion criteria
All studies reporting clinical or radiological outcomes of any type of non-operative management option for symptomatic talar OLTs	Presence of other lower extremity diagnoses other than talar OLT and associated OLT injuries
Primary and non-primary OLTs	< 5 patients included in the study
Level 1–4 studies	Level 5 evidence
Published in a peer-reviewed journal	Studies with overlapping patient groups
Full-text available in English	

### Eligibility criteria and study selection

All studies including title and abstract were independently screened by two independent researchers (JD, KL). When there was no agreement, assessment by an independent third investigator (GK) would be decisive for inclusion or exclusion. Subsequently, full text articles of the initial included studies were viewed for eligibility based on the same in- and exclusion criteria. Again, in case of any conflict, a third author (GK) was consulted for the final decision. The exact inclusion and exclusion criteria are presented in Table 1. No restrictions on age of the included patients were applied.

### Quality assessment

Risk of Bias and quality of the included articles was assessed by two reviewers (TB and JD) with the use of the Methodological Index for Non-Randomized Studies (MINORS) criteria [37]. Each included study was graded by 2 independent reviewers (JD, TB). When there was no agreement, assessment by a third independent investigator (GK) would be decisive.

### Data extraction and pooling

Two reviewers independently collected and cross-checked all the data (KL, TB). The following study characteristics data were collected: authors, title, type of study, level of evidence of study, year of publication, clinical scoring systems used, treatment type applied, indications for non-operative treatment, mean and range of follow-up time. Patient data extracted from the studies included number of patients, mean age, sex, number of ankles, number of patients who converted to surgery including the rate of conversion to surgery, duration of symptoms, clinical outcomes, radiological outcomes and reported success of treatment. In case of several follow-up reports, the latest report is extracted.

### Statistical and data analysis

Clinical outcomes were defined as successful when a good or excellent clinical result at follow-up was reported in a qualitative manner or when at the latest follow-up of a post-operative American Orthopaedic Foot and Ankle Society (AOFAS) score at or above 80 was reached [19]. When clinical outcomes were based on other clinical scoring systems, outcomes reported as good or excellent were considered as clinical success. Subsequently, clinical success rates were calculated per study. A simplified pooling method was used to combine data from different studies using corresponding methodologies to provide clinical success rate results for the overall non-operative treatment group and to provide separate pooled results for the clinical success rates of different subtypes of non-operative treatment options. Ninety-five percent binomial proportion confidence intervals for the success percentages of each study and the pooled studies will be calculated with the Wilson score interval [5]. Radiological changes over the course of conservative treatment were assessed either considering local OLT changes and/or overall ankle joint changes. Pooling of radiological results was exclusively performed whenever the same radiological method was used (i.e., computed tomography, magnetic resonance imaging, and plain radiographs). Whenever possible, pooled rates including 95% confidence intervals were calculated for the following outcomes: rate of unchanged radiological presentation, rate of radiological progression of the lesion, and rate of healing of the lesion.

## Results

### Search results

**Fig. 1:**
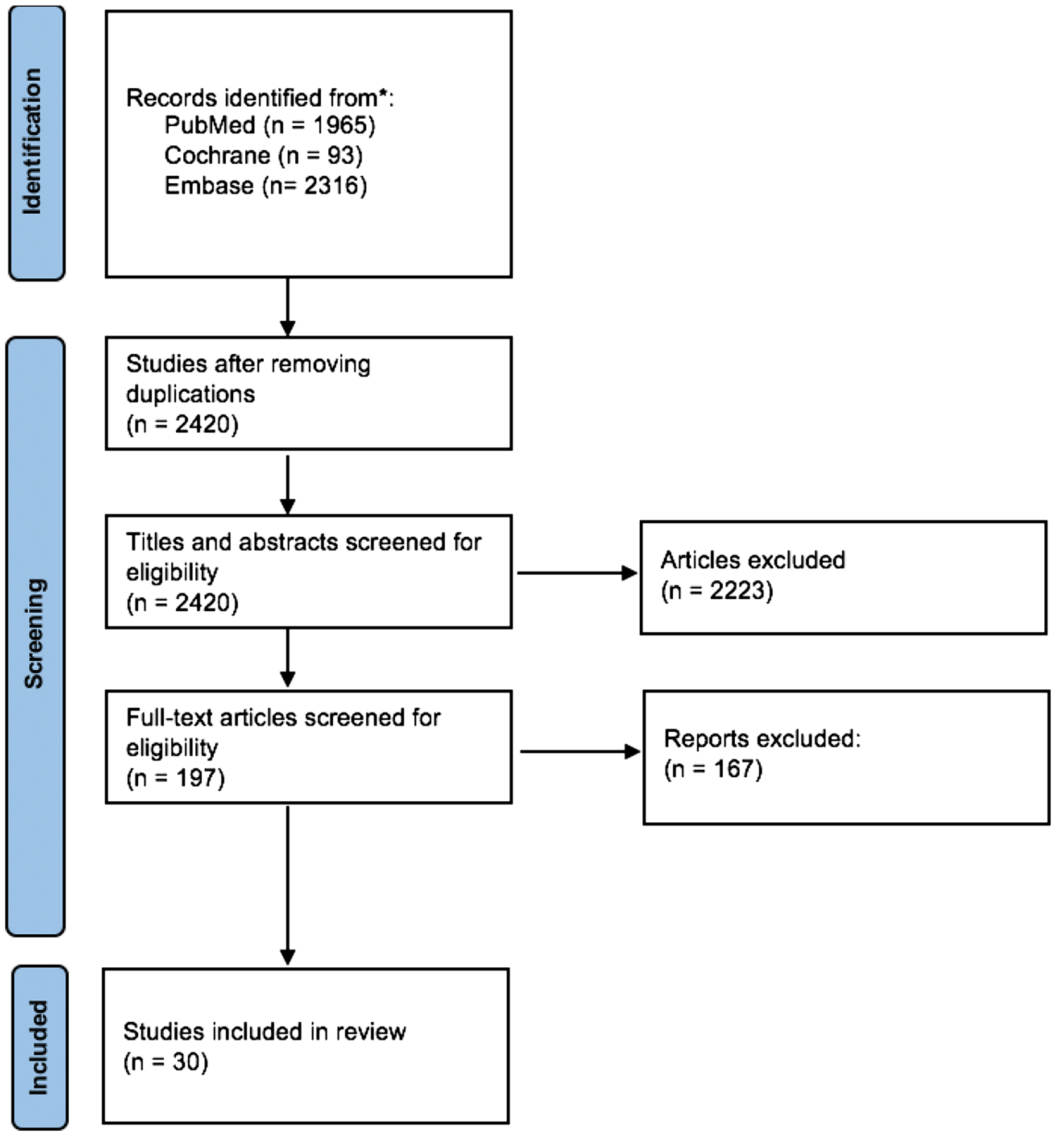
Preferred Reporting Items for Systematic Reviews and Meta-Analysis (PRISMA) flowchart of the performed screening process

**Fig. 2:**
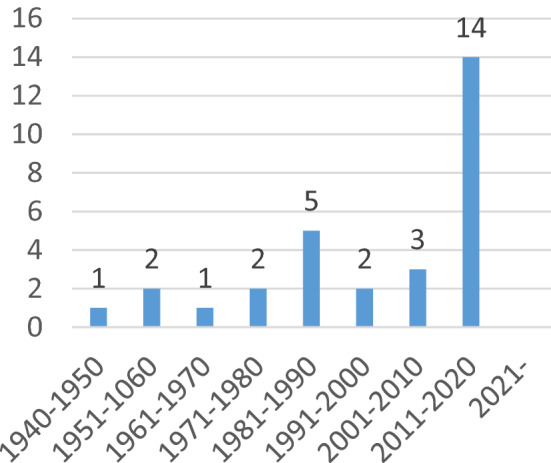
Years of publication of the included studies

The initial search yielded a total of 1824 articles. After the screening of title and abstract, a total of 1625 articles were excluded based on the in- and exclusion criteria. Finally, a total of 30 articles were included (Fig. 1). There were no conflicts during the process. The years of publication of the included studies are summarized in Fig. 2. Twenty-seven studies were retrospective series and only 3 studies had a prospective data registration. All general, clinical and radiological data are presented in Appendix 1.

### Characteristics of included studies

Thirteen articles reported on only (initial) non-operatively treated patients. Seventeen articles reported on both non-operative and surgical management. The total number of initially non-operatively treated patients in the included studies was 868. The median follow-up of the included studies was 37 months, ranging from 3 to 288. The mean age was 33.8 (9.0–56.08). For 626 patients for whom gender was reported, 336 were male and 290 were female. Side of the lesion was reported for 523 lesions, 332 were right sided and 191 were left sided. Location of the lesion on the talus was reported for 592 lesions, 478 lesions were medial, 107 lateral and 7 central on the talus. Only 14 patients, distributed over 3 studies, had non-primary lesions, and no separate outcomes were reported on these patients [23, 27, 28].

Seven articles reported on a post-treatment Visual Analogue Scale (VAS) [8] pain score, with a mean ranging from 0–5.4 [1, 18, 23, 28, 35]. Measuring the treatment effect with the AOFAS score was used in eleven studies with a post-treatment score ranging from 68.2 to 98 [1, 15, 18, 20, 21, 23, 27, 28, 32, 35, 42]. An overview of all study characteristics, clinical and radiological outcomes is presented in Appendix 2.

### Methodological quality

After independent grading and discussion by 2 reviewers (JD, TB), full consensus on methodological quality was reached. The results of the MINORS score for the included studies are presented in Appendix 2.

#### Indications

The indications for non-operative treatment were mentioned in 40% (12/30) of the included studies. Stage 1 or 2 lesions according to the Berndt and Harty classification [17], stage 1, 2 or 3 lesions [1, 7], stage 1, 2, 3 or 4 lesions [30], stage 5 lesions [36], asymptomatic or minimally symptomatic lesions [20, 34, 45], failure of other primary non-operative strategies [23, 28], or minimal activity on SPECT/CT-scans [27]. Additionally, one study reported the lack of justification of surgery for the intensity of symptoms [31].

#### Clinical outcomes

The overall pooled success rate was 45% (168/372; 95%CI 40–50%). Conversion to surgery after initial non-operative management was described in 11 studies yielding a total pooled conversion to surgery rate of 46% (184/400; 95%CI 41–51%). The pooled clinical outcomes are presented in Table 2. If the provided data in selected articles do not include information for conservatively treated patients separately, information is not presented in this table.

**Table 2:** Clinical outcomes

Subtype of treatment		Study	Follow-up time (months, range)	Clinical success rate	Conversion to surgery	Description success
Adjustment of activities		Seo et al. [35]	72 (36–120)	NA	11% (5/56; 95%CI 5–21%)	
		Heyse et al. [15]	NA (NA)	NA	60% (45/75; 95%CI 49–70%)	
Weightbearing restrictions		Flick and Gould [13]	NA (NA)	0% (0/8; 95%CI 0–32%)	63% (5/8; 95%CI 31–68%)	“Excellent”, “Good”, “Fair”, “Poor” based on a self-developed questionnaire
		Huylebroek et al. [17]	NA (NA)	55% (12/22; 95%CI 35–73%)	NA	“Good”: No pain, able to perform all activities or the same level of sports
		Ibanez et al.[18]	NA (NA)	NA	NA	
		Meftah et al. [27]	36 (NA)	NA	NA	
Injectables		Akpancar and Gül [1]	12 (NA)	90% (44/49; 95%CI 78–96%)	NA	“Excellent”: If there was no ankle pain after daily activities or sport activities “Good”: Pain levels <50% of the original ankle pain
PRP OR Prolotherapy						
99mTc-MDP		Liu et al. [23]	7 (6–15)	NA	NA	
Hyaluronate		Gesu et al. [24]	3 (NA)	NA	NA	
PRP OR Hyaluronate		Mei-Dan et al. [28]	7 (NA)	NA	NA	
Other		Berndt et al. [3]	NA (NA)	10% (2/21; 95%CI 3–29%)	67% (14/21; 95%CI: 45–83%)	“Good”: Freedom of symptoms or if only slightly annoying.
		Davidson et al. [11]	20 (0.5–100)	18% (3/17; 95%CI 6–41%)	53% (9/17; 95%CI 31–74%)	Literally mentioned “Good” or “Very good”
		Blom and Strijk [4]	NA (NA)	20% (9/44; 95%CI 11–35%)	30% (13/44; 95%CI 18–44%)	“Good”: Entirely or almost entirely free from symptoms
		McCullough and Venugopal [26]	NA (NA)	83% (5/6; 95%CI 44–97%)	NA	“Excellent”: No symptoms related to ankle “Good”: Occasional ankle discomfort that does not limit normal activity
		Thompson and Loomer [40]	NA (NA)	0% (0/3; 95%CI 0–56%)	NA	“Good”: No pain, no activity restrictions,normal physical exam and a normal plain radiograph
		Pettinne and Morrey [30]	NA (NA)	46% (17/37; 95%CI 31–62%)	NA	“Good”: Asymptomatic
		Zinman et al. [45]	NA (NA)	13% (4/31; 95%CI 5–29%)	71% (22/31; 95%CI 53–84%)	“Excellent”: Returned to regular activity and no limitation or pain while running or walking “Good”: Not limited in walking, but who had occasional pain
		Wester et al. [43]	288 (84–432)	NA	NA	
		Higuera et al. [16]	NA (NA)	NA	9% (1/11; 95%CI 2–38%)	
		Shearer et al. [36]	38 (17–85)	54% (19/35; 95%CI 38–70%)	17% (6/35; 95%CI 8–33%)	“Excellent”: Symptoms are gone or greatly improved and no sport limitations “Good”: 1) Symptoms are gone or greatly improved and some limitations in sports or 2) Slightly improved symptoms and no sport limitations
		Letts et al. [22]	NA (NA)	38% (9/24; 95%CI 21–57%)	54% (13/24; 95%CI 35–72%)	“Good”: Occasional symptoms without loss of function
		Perumal et al. [29]	12 (NA)	16% (5/31; 95%CI 7–33%)	39% (12/31; 95%CI 24–56%)	Complete clinical and radiological healing
		Lam et al. [21]	30.5 (11–63)	100% (6/6; 95%CI 61–100%)	NA	“Excellent” or “Good” based on the Higuera classification
		Reilingh et al. [33]	NA (NA)	NA	92% (34/37; 95%CI 79–97%)	
		Rehnitz et al. [32]	NA (NA)	69% (11/16; 95%CI 44–86%)	NA	AOFAS > 80
		Weigelt et al. [42]	168 (132–240)	100% (22/22; 95%CI 85–100%)	NA	Good or excellent on the AOFAS score
		Pooled result		45% (168/372; 95%CI 40–50%)	46% (184/400; 95%CI 41–51%)	

Methylene Diphosphonate

*AOFAS* American Orthopedic Foot and Ankle Outcome Score, *NA* Not Available, 99mTc-MDP: Technetium-99 m

### Radiological outcomes

#### Plain radiographs radiological outcomes

Definitions of healing on plain radiographs being used in the included studies were decrease in size of the lesion (width, depth or length) or the description of any signs of decrease in the lesion size. Definitions of unchanged lesions being used in the included studies were unchanged lesions, no signs of ossification, unhealed lesions or still visible lesions. The use of a plain radiograph to assess the radiological changes of the lesion was described in 5 articles [21, 26, 29, 34, 43]. As assessed with a plain radiograph, radiological deterioration was observed in 84% (76/91; 95%CI 75–90%) of the patients. More specifically, the pooled healing rate was 31% (28/91; 95%CI 22–41%). An unchanged lesion was detected on plain radiographs in 53% (48/91; CI 43–63%) of the patients, and a progression of the defect was seen in 23% (21/91; 95%CI 16–33%) of the patients. Osteoarthritic changes, i.e., overall ankle joint changes, as assessed with plain radiographs were reported in 5 studies [20, 26, 35, 36, 42]. Pooling of patients with progression of osteoarthritis of the ankle showed a rate of 9% (185/204; 95%CI 6–14%) as assessed with the van Dijk osteoarthritis scale [41].

#### Computed tomography radiological outcomes

The use of a CT scan to assess radiological outcomes was described in 4 articles [23, 35, 36, 43]. Healing of lesions on CT scans were described if a decrease in size (width, depth or length) was observed or if CT scans were rated as ‘’normal radiological findings’’. Radiological outcomes based on a Computed Tomography (CT) scan were described in 4 studies. No radiological deterioration was observed in 89% (116/131; 95%CI 82–93%) of the patients. More specifically, the pooled healing rate was 13% (17/131; 95%CI 8–20%) [23, 35, 36, 43], the unchanged radiological rate was 76% (99/131; 95%CI 68–82%) of the patients, and progression of the defect was observed in 11% (15/131; 95%CI 7–18%) of the patients.

#### MRI radiological outcomes

Changes on MRI were observed in case of change in stage on the Andersen Scale. The use of an MRI scan to assess radiological outcomes was described in 1 article [20]. No radiological deterioration of the lesion was observed in 88% (44/50; 95%CI 76–94%) of the patients. Healing was observed in 4% (2/50; 95%CI 1–13%) of the patients, no change of the lesion was observed in 84% (42/50; 95%CI 71–92%) of the patients, and a progression of the lesion was observed in 12% (6/50; 95%CI 6–24%) of the patients [20].

### Types of non-operative treatment

#### Adjustment of activities

Solely adjustment of activities, also called a “benign neglect” was described in two studies including a total number of 357 patients of which 218 patients were treated with activity adjustment [15, 35]. Success rates were not reported in this subgroup. A total of 39% (51/131; 95%CI 31–47%) of the patients were converted to surgery. Seo et al. [35] described a mean pre-treatment VAS pain of 3.8 and a mean post-treatment VAS pain of 0.9 of 142 patients that were treated with activity adjustment. Heyse et al. [15] reported a mean post-treatment AOFAS ankle hindfoot score of 68.2 and an Olerud/Molander score of 90.6 in 30 patients. Only Seo et al. [35] reported radiological outcomes with healing of the lesion in 6% (5/83; 95%CI 3–13%), unchanged lesion in 83% (69/83; 95%CI 74–90%) and progression of the lesion in 11% (9/83; 95%CI 6–19%). Additionally, 0% (0/0; 95%CI 0–3%) showed progression of osteoarthritis.

#### Weight bearing restrictions

Solely weight bearing restrictions or in combination with a cast was applied in 4 studies including a total of 51 patients [13, 17, 18, 27]. The time of cast immobilization ranged between 3 and 8 weeks. The pooled success rate in this group was 55% (12/22; 95%CI 34–73%). The calculated rate of conversion to surgery was 63% (5/8; 95%CI 31–86%).

Ibanez et al. [18] reported a mean pre-treatment AOFAS ankle hindfoot score of 58 and a mean post-treatment score of 74.8. The mean VAS score in this study improved from 9 to 5.4. Meftah et al. [27] reported a mean AOFAS post-treatment score of 87.3. Lam et al. [21] reported a mean post-treatment AOFAS hindfoot score of 90. None of the studies in this subgroup reported on radiological outcomes.

#### Injectables

Solely Injectables were applied in four articles and included the following types: Platelet Rich Plasma (PRP), HA, sodium hyaluronate, prolotherapy and TC-methylene diphosphonate with herbal fumigation [1, 23, 24, 28]. The total number of patients was 175 and the median time of follow-up was 5 months. One study reported success rate of 90% (44/49; 95%CI 78–96%) [1]. Conversion to surgery was not reported in any of the selected studies in this group. The number of injections and time intervals are described in Appendix 2. Mei-Dan et al. [28] reported an improvement on the VAS (pain) scale from 5.6 to 3.1 in the HA group and from 4.1 to 0.9 in the PRP group. The AOFAS improved from 66.4 to 78.3 in the HA group and from 68 to 92.5 in the PRP group. Liu et al. [23] reported an improvement from 3.05 to 1.85 on the VAS (pain) scale and from 68.66 to 85.40 on the AOFAS score. Gesu et al. [24] reported an improvement from 52 to 98 on the AOFAS scale.

Akpancar and Gül [1] reported an improvement on the VAS pain scale from 7.15 to 1.30 in the prolotherapy group and from 7.73 to 1.41 in the PRP group. AOFAS improved from 38.48 to 89.44 in the prolotherapy group and 30.09 to 87.77 in the PRP group.

Only Liu et al. [23] reported on radiological outcomes and observed a decrease in cyst average from 8.1 to 4.7 mm in diameter.

#### Other

Shearer et al. [36], Berndt et al. [3] and Pettine et al. [30] reported on different types of non-operative management in one study without reporting results on each subgroup. Non-operative management in these studies includes weight bearing restrictions, use of NSAIDs, physiotherapy, casts and braces.

Combination of non-operative modalities were reported in five studies and were as follows: restricted sports activities and physical therapy, taping or treatment with a plaster cast, cast immobilization and restrictions of activities, cast immobilization and protected weight bearing, cast immobilization and activity modification, activity restriction and physical therapy [21, 22, 27, 29, 33]. Eight articles described the use of a brace or strapping [3, 13, 29–31, 33, 36, 43]. One article described the use of shockwave therapy as non-operative management [39].

## Discussion

The most important finding of the present study is that non-operative treatment for osteochondral lesions of the talus is clinically effective in 45% of the patients. No evidence was identified that one of the subtypes of non-operative management protocols were superior or inferior to one another from a clinical or radiological perspective.

It is to be stated that non-operative treatment is recommended as first treatment in line after the initial diagnosis of an OLT as a result of our findings [12]. The used strategies in non-operative treatment were heterogeneous as they differed widely in the included articles. Four articles used a so called “benign neglect’’ or modification of the activities [15, 35, 36, 40]. Seo et al. [35] stated that good clinical results were obtained with a mean AOFAS of 93 points at a long-term follow-up. Another interesting finding from the study of Seo et al. [35] was that the average VAS score of pain at baseline was 3.8, suggesting that patients did not have major complaints at the onset of treatment. Despite that a lower level of complaints might be an indication for the choice of non-operative treatment, other indications were not mentioned in any of the studies reporting on solely activity restrictions or a “benign neglect’’.

Weightbearing restrictive treatment modalities were also frequently reported non-operative treatment options as four studies were included in our systematic review on this particular treatment modality [13, 17, 18, 27]. Periods of immobilization in the included studies ranged from 3 to 8 weeks. It was found that the success rates having been found in the studies on immobilization were around 50% [17, 30]. There was no superiority observed concerning clinical outcomes with longer immobilization periods. Despite this promising result, no radiological results can demonstrate improvement of the lesion due to immobilization.

Two articles described the use of injectables by injecting hyaluronic acid or 99 m TC-methylene diphosphonate. Liu et al. [23] noticed substantial clinical progression in terms of the AOFAS and VAS scores after injection of TC-methylene diphosphonate with herbal fumigation. However, this intervention is totally new and the literature on this topic is scarce which made it impossible to draw conclusions on the working mechanism and potential effect of this treatment strategy. Hyaluronic acid injections showed its potential in improving the clinical symptoms. This may be due to the chondroprotective and anti-inflammatory effect which limiting degenerative changes coming along with the osteochondral defect [38]. Another injectable that was studied was Platelet Rich Plasma [1, 28]. These injections showed considerable improvements when considering the pain scores as measured with the VAS scale. However, it should be mentioned that these studies were conducted in small populations including relatively low number of patients. Moreover, the reported results were assessed at short-term follow-up. As such, one could state that specific conclusions considering the efficacy of injection therapy cannot be made due to the low level of evidence originating from these studies.

One can state that the methodological quality of injection therapies for OLTs was considered low, and the potential and indications of injection therapies as part of non-operative treatment for OLTs need to be further investigated in future double-blind placebo-controlled prospectively randomized studies.

In addition to different types of non-operative management, duration of the non-operative management is an important factor to analyze. Activity restrictions or cast immobilization were applied for a period ranging from 3 to 8 weeks. After a period of activity restriction, casting, physiotherapy or a combination of all, patients and clinicians must decide whether non-operative management meets their expectations of the treatment or if surgical treatment is indicated. The current literature suggests considering surgical management after trying non-operative management for at least 6 months. This is corresponding with literature included in this study [12]. Additionally, the literature concerning osteochondral lesions of the knee suggests that lesions can heal within a time of six months which seems to confirm that 6 months is a proper duration [2].

The conversion to surgery rate emphasizes that a certain part of the population has no indication for direct surgical treatment. However, based on the results on this review, it is difficult to define clear indications for non-operative management. Described indications varied widely in the included studies in terms of lesion characteristics and level of complains. It was therefore not possible to analyze if specific indications were superior to others. One of the indications that need to be discussed is the justification of a surgical treatment. In several studies it is stated that patients avoided surgery or that they stated that their symptoms simply did not justify their complaints [17]. This reveals that the decision-making process for non-operative management is highly important in a patient group who can accept a lower functional status with minor complains [12]. Based on the statement of Dobrowski et al. [12] and the results of present review non-operative management is advocated for at least 6 months which can be extended based on a shared-decision-making process and regular clinical and radiological follow-ups.

Concerning the radiological outcomes which were analyzed in the present study, one can note that radiological healing was assessed in the studies of Perumal et al. [29] and Wester et al. [43] having shown a healing rate of 18% and 69%, respectively. This healing rate can be considered relatively high compared to other studies [34, 36]. These healing rates can be explained by the fact that Perumal et al. [29] and Wester et al. [43] included patients from a pediatric population as it is known that patients with open growth plates have a higher healing potential [6, 14]. However, it remains unclear to what extent precisely a casting and weight bearing restriction protocol may support this healing.

It should, however, also be noted that a selective group of 10% developed arthritic changes in a relatively short period. This must aware clinicians in the risk of possible deterioration when starting conservative management. It is therefore highly recommended to have an intensive follow-up including CT-scans and physical examinations after the start of conservative management to change the treatment path and avoid irreversible damage. To identify patients benefitting from non-operative management, prospective studies on different non-operative treatment modalities are needed. These studies need to include radiological follow-up too as it enables caretakers and patients to see which impact non-operative management has on the radiological characteristics of the lesion.

Despite the discrepancy between the healing rate and the pooled success rate of non-operative treatment, it must be emphasized that the majority of the lesions do not progress over time from a radiological perspective nor showed development of osteoarthritis. The latter is an important fact in the provision of information to patients to manage their expectations.

This review has a number of strengths. First, this review was pre-registered in the PROSPERO database [9]. It must also be stated that the thorough reference selection and quality assessment of the included studies can be considered strengths of the study. Moreover, to the best of our knowledge, this is the first review summarizing different types of non-operative management including its clinical and radiological outcomes. As such, the clinical relevance of the present study entails that the summary of a comprehensive overview of the different types of non-operative treatment modalities including their clinical- and radiological outcomes will aid and improve the decision-making process.

Besides its strengths, there were a number of limitations concerning the present review. The included studies were mostly retrospective in nature and published before 1990 causing a high heterogeneity among the studies in terms of success definition, included population, follow-up moments and indications. Due to this heterogeneity, results need to be interpreted with caution as there is a high chance of indication bias. One of the heterogeneous factors was the difference in the use of clinical outcomes. Clinical success was defined as good or excellent outcomes, or an AOFAS score > 80. However, “good’’ or “excellent’’ outcomes leaves space for interpretation. The following criteria were used in the included studies to classify the outcome of the treatment: conversion to surgery, Higuera classification [16], clinical symptoms such as pain and functional results such as activity restrictions. It can be logically argued that such a wide range of definitions may lead to bias of the results. Secondly, different patient selections took place for the indication of conservative management introducing a potential selection bias. Additionally, it was found that 2% of the lesions were of non-primary (i.e., secondary or tertiary) nature, and, as, such, it is not expected that this may influence the outcomes of the present study.

## Conclusion

Non-operative management for osteochondral lesions of the talus yielded an overall clinical success rate of 45%. Radiological outcomes showed that 11% of the patients deteriorate over time considering whole ankle joint osteoarthritic changes and considering focal radiological deterioration to the OLT. No evidence was found that one of the subtypes of non-operative management protocols were superior or inferior to one another.

## Supplementary Information

Below is the link to the electronic supplementary material.

Supplementary file1 (DOCX 34869 KB)

## Data Availability

Dataset that has been used for analysis is available from the corresponding author.
