# Supplementary material for: Non-operative management for osteochondral lesions of the talus: a systematic review of treatment modalities, clinical- and radiological outcomes
Source: Knee Surg Sports Traumatol Arthrosc. 2023 Apr 16;31(8):3517–27. doi: 10.1007/s00167-023-07408-w (PMC10356662; doi:10.1007/s00167-023-07408-w)

Appendix 1. MINORS Score. MINORS score of included articles. 0= not reported, 1= reported but inadequately, 2=adequately reported

| Study | A clearly stated aim | Inclusion of consecutive patients | Prospective collection of data | Endpoint appropriate to the aim of the study | Unbiased assessment of the study endpoint | Follow-up period appropriate to the aim of the study | loss of follow-up less than 5% | Prospective calculation of the study size | An adequate control group | Contemporary group | Baseline equivalence of groups | Adequate statistical analysis | Total |
| --- | --- | --- | --- | --- | --- | --- | --- | --- | --- | --- | --- | --- | --- |
| Ray and Coughlin [33] | 0 | 0 | 0 | 0 | 2 | 0 | 0 | 0 | N.A. | N.A. | N.A. | N.A. | 2/16 |
| Roden et al. [36] | 0 | 0 | 0 | 0 | 2 | 0 | 0 | 0 | N.A. | N.A. | N.A. | N.A. | 2/16 |
| Berndt et al. [3] | 0 | 0 | 0 | 2 | 2 | 2 | 0 | 0 | N.A. | N.A. | N.A. | N.A. | 6/16 |
| Davidson et al. [12] | 0 | 0 | 0 | 0 | 2 | 0 | 0 | 0 | N.A. | N.A. | N.A. | N.A. | 2/16 |
| Blom and Strijk [4] | 0 | 0 | 0 | 0 | 2 | 0 | 0 | 0 | N.A. | N.A. | N.A. | N.A. | 2/16 |
| McCullough and Venugopal [28] | 2 | 0 | 0 | 2 | 2 | 2 | 0 | 0 | N.A. | N.A. | N.A. | N.A. | 8/16 |
| Thompson and Loomer [42] | 0 | 0 | 0 | 2 | 2 | 2 | 0 | 0 | N.A. | N.A. | N.A. | N.A. | 6/16 |
| Huylebroek et al. [18] | 0 | 0 | 0 | 2 | 2 | 2 | 0 | 0 | N.A. | N.A. | N.A. | N.A. | 6/16 |
| Flick and Gould [14] | 0 | 0 | 0 | 2 | 2 | 2 | 0 | 0 | N.A. | N.A. | N.A. | N.A. | 6/16 |
| Pettine et al. [32] | 2 | 0 | 0 | 2 | 2 | 2 | 0 | 0 | N.A. | N.A. | N.A. | N.A. | 8/16 |
| Zinman et al. [47] | 0 | 0 | 0 | 2 | 2 | 2 | 0 | 0 | N.A. | N.A. | N.A. | N.A. | 6/16 |
| Wester et al. [45] | 0 | 0 | 0 | 0 | 2 | 0 | 0 | 0 | N.A. | N.A. | N.A. | N.A. | 2/16 |
| Higuera et al. [17] | 2 | 0 | 0 | 2 | 2 | 2 | 0 | 0 | N.A. | N.A. | N.A. | N.A. | 8/16 |
| Shearer et al. [38] | 2 | 0 | 0 | 2 | 2 | 2 | 0 | 0 | N.A. | N.A. | N.A. | N.A. | 8/16 |
| Letts et al. [24] | 2 | 0 | 0 | 2 | 2 | 2 | 0 | 0 | N.A. | N.A. | N.A. | N.A. | 8/16 |
| Perumal et al. [31] | 2 | 0 | 0 | 2 | 2 | 2 | 0 | 0 | N.A. | N.A. | N.A. | N.A. | 8/16 |
| Meftah et al. [29] | 2 | 0 | 0 | 2 | 1 | 2 | 0 | 0 | N.A. | N.A. | N.A. | N.A. | 7/16 |
| Mei-Dan et al. [30] | 2 | 0 | 2 | 2 | 2 | 2 | 2 | 0 | 2 | 2 | 2 | 2 | 20/24 |
| Lam and Siow [22] | 2 | 0 | 0 | 2 | 2 | 2 | 0 | 0 | N.A. | N.A. | N.A. | N.A. | 8/16 |
| Reilingh et al. [35] | 2 | 0 | 0 | 2 | 2 | 2 | 2 | 0 | N.A. | N.A. | N.A. | N.A. | 10/16 |
| Ibanez et al. [19] | 2 | 0 | 0 | 2 | 2 | 2 | 0 | 0 | N.A. | N.A. | N.A. | N.A. | 8/16 |
| Heyse et al. [16] | 2 | 0 | 0 | 2 | 1 | 2 | 0 | 0 | N.A. | N.A. | N.A. | N.A. | 7/16 |
| Thiele et al. [41] | 0 | 0 | 0 | 2 | 2 | 0 | 0 | 0 | N.A. | N.A. | N.A. | N.A. | 4/16 |
| Klammer et al. [21] | 2 | 0 | 0 | 2 | 2 | 2 | 0 | 0 | N.A. | N.A. | N.A. | N.A. | 8/16 |
| Gesu et al. [26] | 2 | 0 | 0 | 2 | 2 | 2 | 0 | 0 | N.A. | N.A. | N.A. | N.A. | 8/16 |
| Liu et al. [25] | 2 | 0 | 0 | 2 | 2 | 2 | 0 | 0 | N.A. | N.A. | N.A. | N.A. | 8/16 |
| Rehnitz et al. [34] | 2 | 0 | 0 | 2 | 1 | 2 | 0 | 0 | N.A. | N.A. | N.A. | N.A. | 7/16 |
| Seo et al. [37] | 2 | 0 | 0 | 2 | 1 | 2 | 2 | 0 | 2 | 1 | 2 | 2 | 14/24 |
| Weigelt et al. [44] | 2 | 0 | 0 | 2 | 2 | 2 | 0 | 0 | N.A. | N.A. | N.A. | N.A. | 8/16 |
| Akpancar and Gül [1] | 2 | 1 | 0 | 2 | 2 | 2 | 0 | 0 | N.A. | N.A. | N.A. | N.A. | 9/16 |

Appendix 2. Search

| **#** | **Searches** |
| --- | --- |
| 1 | osteochondritis dissecans/ or (osteochondritis dissecans or osteochondrosis dissecans or osteochondrolysis or OCD or OLT).ti,ab,kw. or ((osteochondral or chondral or transchondral or cartilage*) and (defect* or lesion*)).ti,ab,kw. |
| 2 | talus/ or (talus or talar* or ankle*).ti,ab,kw. |
| 3 | 1 and 2 |
| 4 | limit 3 to conference abstract status |
| 5 | 3 not 4 |
| 6 | animal/ not human/ |
| 7 | 5 not 6 |
|  | Initial search: 04-12-2018; update 1: 21-09-2020; update 2: 10-08-2022 |

Appendix 3. Oversight of all outcomes


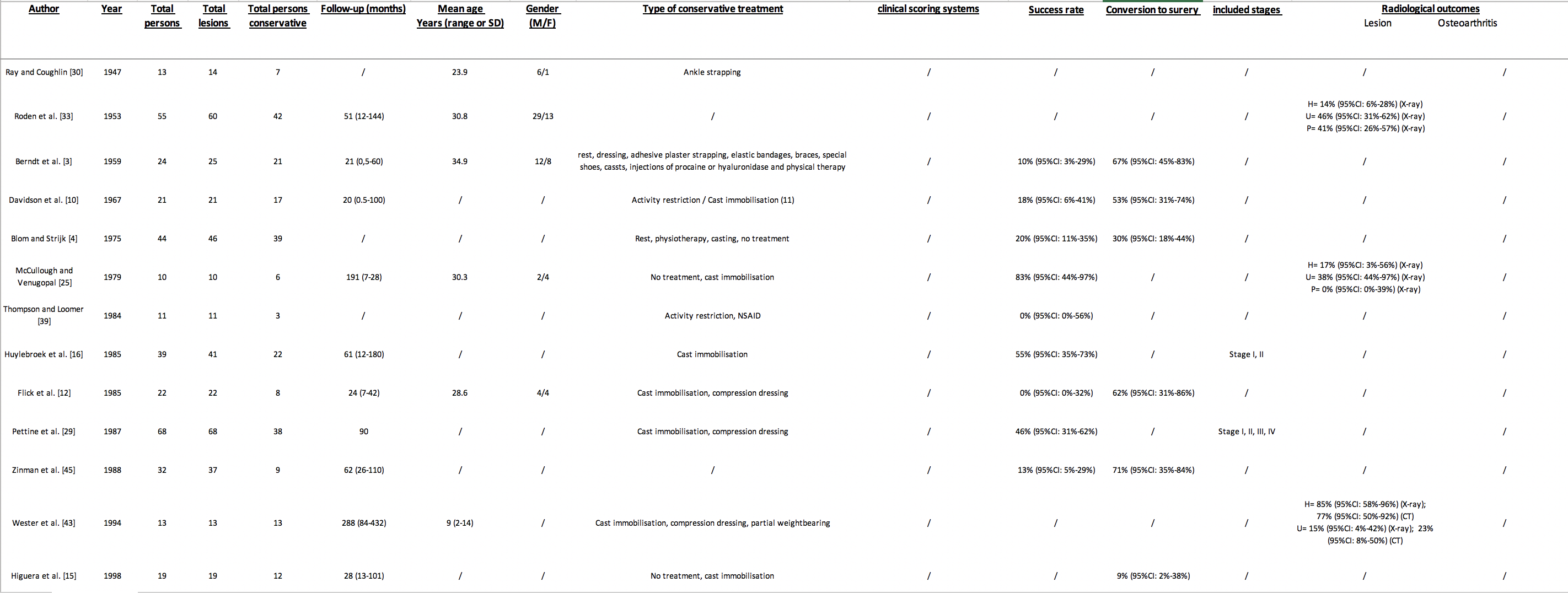


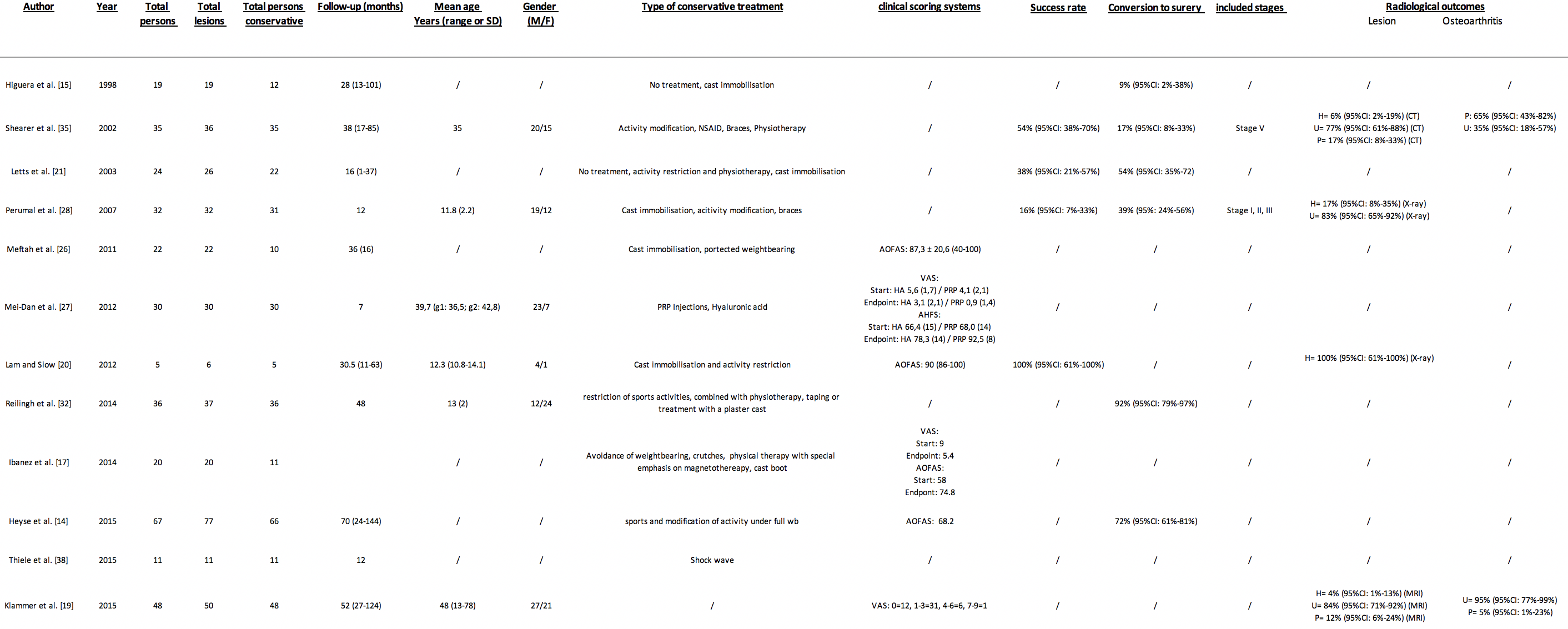


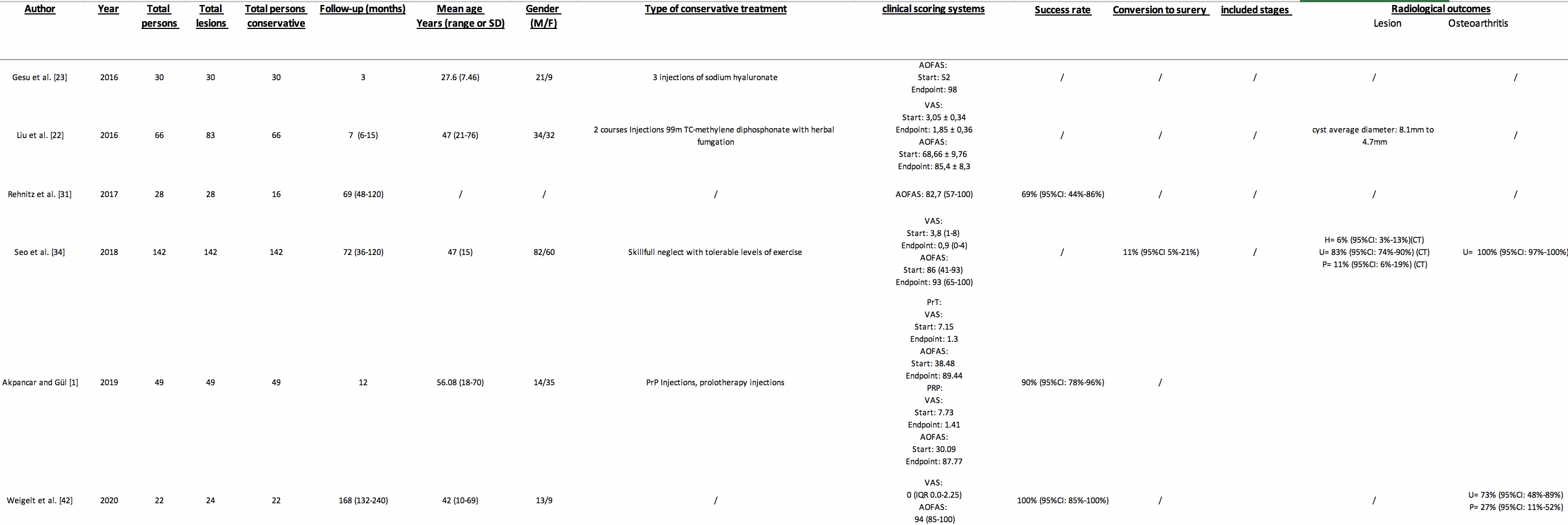

Supplement: Supplementary file 1 — Supplementary file1 (DOCX 34869 KB) [file 167_2023_7408_MOESM1_ESM.docx]
